# A deep learning model for predicting COVID-19 ARDS in critically ill patients

**DOI:** 10.3389/fmed.2023.1221711

**Published:** 2023-07-25

**Authors:** Yang Zhou, Jinhua Feng, Shuya Mei, Ri Tang, Shunpeng Xing, Shaojie Qin, Zhiyun Zhang, Qiaoyi Xu, Yuan Gao, Zhengyu He

**Affiliations:** Department of Critical Care Medicine, Renji Hospital, School of Medicine, Shanghai Jiaotong University, Shanghai, China

**Keywords:** COVID-19, ARDS, deep learning, artificial intelligence, computated tomography

## Abstract

**Background:**

The coronavirus disease 2019 (COVID-19) is an acute infectious pneumonia caused by a severe acute respiratory syndrome coronavirus 2 (SARS-CoV-2) infection previously unknown to humans. However, predictive studies of acute respiratory distress syndrome (ARDS) in patients with COVID-19 are limited. In this study, we attempted to establish predictive models to predict ARDS caused by COVID-19 via a thorough analysis of patients' clinical data and CT images.

**Method:**

The data of included patients were retrospectively collected from the intensive care unit in our hospital from April 2022 to June 2022. The primary outcome was the development of ARDS after ICU admission. We first established two individual predictive models based on extreme gradient boosting (XGBoost) and convolutional neural network (CNN), respectively; then, an integrated model was developed by combining the two individual models. The performance of all the predictive models was evaluated using the area under receiver operating characteristic curve (AUC), confusion matrix, and calibration plot.

**Results:**

A total of 103 critically ill COVID-19 patients were included in this research, of which 23 patients (22.3%) developed ARDS after admission; five predictive variables were selected and further used to establish the machine learning models, and the XGBoost model yielded the most accurate predictions with the highest AUC (0.94, 95% CI: 0.91–0.96). The AUC of the CT-based convolutional neural network predictive model and the integrated model was 0.96 (95% CI: 0.93-0.98) and 0.97 (95% CI: 0.95–0.99), respectively.

**Conclusion:**

An integrated deep learning model could be used to predict COVID-19 ARDS in critically ill patients.

## Introduction

The coronavirus disease 2019 (COVID-19) is an acute infectious pneumonia caused by a severe acute respiratory syndrome coronavirus 2 (SARS-CoV-2) infection (1). Evidence has shown that 33% of COVID-19 patients are at high risk of progressing into severe cases, which are accompanied by increasing mortality and morbidity (2, 3). Moreover, severe SARS-CoV-2 infection may directly lead to acute respiratory distress syndrome (ARDS), and the manifestations could be viewed as a combination of pneumonia and ARDS (4).

Although significant advances have been made in understanding and managing ARDS, the morbidity and mortality of patients diagnosed with ARDS still remain high (5). Unfortunately, the benefits of different therapies for established ARDS are limited (6–8). Since then, the paradigm for the management of ARDS has been shifted from treatment to prevention. Identification of patients at high risk of ARDS is important for clinicians to implement effective, preventive therapies to reduce the burden of ARDS. It is reported that the median time from the onset of COVID-19 symptoms to intubation is 8.5 days when COVID-19 ARDS occurs (9). There have been several studies focusing on the early prediction of ARDS, which described well-known risk factors associated with ARDS (10–12). However, COVID-19 ARDS is a serious complication of COVID-19, which has different clinical features from pre-COVID-19 ARDS (13). Hence, a clinical tool tailored for predicting COVID-19 ARDS is urgently needed.

In recent years, artificial intelligence (AI) has emerged as a promising tool in the medical field. The remarkable advantage of artificial intelligence in handling massive data could help with disease diagnostics and prognostics, radiographic recognition, and personalized treatment, etc. (14). During the COVID-19 pandemic, first-hand CT and clinical datasets helped clinicians make decisions and better understand the viral infection. For example, elevated levels of inflammatory cytokines and a reduction of T-cell subsets are closely related to COVID-19 pneumonia (15). The radiology features of COVID-19 pneumonia include a peripheral distribution of opacification, frosted glass opacities, and vascular thickening and enlargement (16). In spite of the distinct features observed in COVID-19 patients, the clinician may find it hard to figure out the underlying correlations between the clinical features and the features of CT slices, hindering the comprehensive understanding of the disease. Here, we aimed to provide a method pooling all the patients' features including CT and clinical features for improving the precision of the prediction of COVID-19 ARDS.

## Methods

This is a retrospective study approved by the institutional Ethics Committees at Shanghai Renji Hospital, and informed patient consent was waived.

### Study patients

All patients admitted to the intensive care unit in Shanghai Renji Hospital between April 2022 and June 2022 were screened for eligibility. Inclusion criteria were as follows: (1) patients who were 18 years old and above; and (2) patients who met the diagnosis of COVID-19 ARDS. Exclusion criteria were as follows: (1) patients who were diagnosed with ARDS within the first day of admission; (2) missing clinical data were more than 20%; and (3) without any CT scan results.

### Diagnosis of COVID-19 ARDS

SARS-CoV-2 infection can be identified by the detection of viral RNA in nasopharyngeal secretions via PCR test. The diagnosis of COVID-19 was confirmed by the patients' clinical history, epidemiological contact, and a positive SARS-CoV-2 test.

The diagnosis of ARDS followed the Berlin definition: (1) requirement of mechanical ventilation and positive end-expiratory pressure or continuous positive airway pressure ≥ 5 cmH_2_O; (2) a certain degree of hypoxemia: severe (PaO_2_/FiO_2_ ≤ 100 mmHg), moderate (PaO_2_/FiO_2_ between 100 mmHg and 200 mmHg), or mild (PaO_2_/FiO_2_ between 200 mmHg and 300 mmHg); and (3) without evidence of pleural effusion, lung collapse, lung nodules, or cardiogenic pulmonary edema from the chest radiography (16). A patient who satisfied the criteria of COVID-19 and ARDS was diagnosed with COVID-19 ARDS.

### Data collection

We collected the first sets of chest CT images and clinical data after the patients' admission to the intensive care unit. The clinical data included demographic information, comorbidity conditions, respiratory support methods, onset symptoms, vital signs at admission, aeration variables, routine blood tests, inflammation tests, biochemical tests, blood coagulation tests, lymphocyte subset tests, and cytokine profile tests. Original CT images both in JPG and DICOM format of the included patients were collected. In this study, we randomly divided the patients into training and validation cohorts in a ratio of 7:3.

### Statistical analysis

The categorical variables were presented as counts and corresponding proportions and were further compared using the chi-square test or Fisher's exact test. The continuous variables were reported as the median and the interquartile range; the Mann–Whitney U-test was applied to compare the differences between the groups. The multivariate logistic regression was performed to figure out the independent risk factors associated with COVID-19 ARDS. A nomogram plot was further established based on the result of the multivariate logistic regression. A two-tailed *P*-value of <0.05 was considered significant. The data analysis in this study was completed via Python version 3.8 and R version 4.0.5.

### The COVID-19 ARDS prediction based on clinical features

Four different machine learning algorithms were implemented to establish the predictive models for COVID-19 ARDS, including logistic regressio*n* (LR), support vector machine (SVM), random forest (RF), and extreme gradient boosting (XGBoost). The training cohort was divided into five partitions, of which four-fifths were used to train the models, and the remaining part was used to validate the models. The hyperparameters of all the models were fine-tuned for the highest area under the receiver operating to avoid the problem of overfitting. We followed two specific rules when searching for the best hyperparameters, which were as follows: (1) the training loss was the lowest after the test of all combinations of hyperparameters; (2) the log loss in the validation cohort was less than –log 0.5 and higher than the training cohort. Grid search with 5-fold cross-validation was applied to search for the most appropriate hyperparameters in the training cohort. Finally, the predictive performance of established models was compared in the validation cohort.

### The labeling of individual CT slices

We first manually labeled 897 slices of 30 patients to train the classification model for individual CT slices. The CT slices were classified into two types: (1) normal CT, in which the image features in lungs were consistent with healthy lungs; (2) abnormal CT, in which image features were associated with COVID-19 pneumonia. Two senior ICU clinicians (ZHand YG) independently labeled individual CT slices. Any disagreements were resolved through discussion. The deep learning framework was based on the architecture of VGG-16, which consisted of 13 convolutional layers and 3 fully connected layers. We further internally validated the classification model and used it to label the remaining 2, 300 CT slices. Finally, every CT slice was classified into a normal CT image or an abnormal CT image.

### The COVID-19 ARDS prediction based on CT images

After the auto-labeling of individual CT slices, we assumed that an abnormal CT slice classified by the model was a positive case. Then, the possibility of being an abnormal CT slice for every CT image was calculated. The 10 most probable abnormal CT slices of a single patient were viewed as the representative CT images and were input into the second VGG-16 network. This convolutional neural network (CNN) allows for the shift from the prediction of COVID-19 ARDS based on individual CT slices to the prediction based on a single patient. The VGG-16 network consists of 1 input layer, 13 convolutional layers, 3 fully connected layers, and 1 output layer. The convolutional layers were used to handle feature extraction and presentation. The pooling layers were used for filtering abundant information under the max-pooling strategy. In the last three output layers, the possibility of being a positive case was calculated for each CT slice. For the individual CT-based prediction, the possibility ranged from 0 to 1, representing a CT slice classified into a normal CT image or an abnormal CT slice. For the single patient-based prediction, the possibility ranged from 0 to 1, representing a patient being predicted to develop COVID-19 ARDS or not.

### The integration of predictions models

The integration of two prediction models based on CT images and clinical data was achieved by the penalized logistic regression algorithm. The L2 regularization of the penalized logistic regression algorithm was used. To be specific, the machine learning model based on clinical features and the CNN model based on CT images individually generated two scores for the prediction of COVID-19 ARDS, which were taken as input features for the penalized logistic regression algorithm. At last, the penalized logistic regression algorithm calculated a prediction score for the COVID-19 ARDS outcome.

### The evaluation of model performance

We randomly divided the patients into the training cohort and the validation cohort in a ratio of 7:3. The overall predictive performance of the integrated model was measured in the test cohort. The receiver operating characteristics (ROC) curve and the confusion matrices of all established predictive models were depicted to compare the performance of the predictive models. A ROC curve is a graphic plot used to illustrate a binary classifier's diagnostic ability as the discrimination threshold varies. It is created by plotting the true-positive rate against the false-positive rate at different discrimination thresholds. The calibration plots were also depicted to assess the predictive performance of all the models.

## Results

### Baseline clinical features of included patients

**Figure 1:**
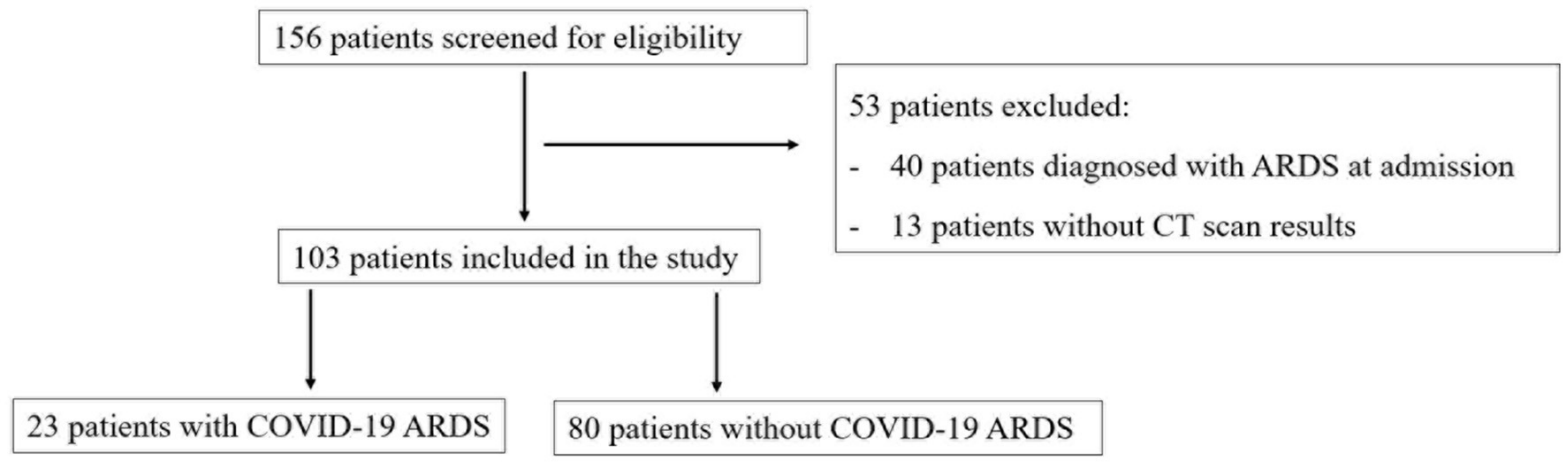
The flowchart of patients' selection.

In total, 103 patients were enrolled in the study after the screening for eligibility, of whom 23 patients (22.3%) developed COVID-19 ARDS. The flowchart of the patients' selection is provided in Figure 1. The baseline clinical features of the included patients are presented in Table 1. There were no missing data in our study.

**Table 1:** Baseline characteristics of included patients.

**Characteristics**	**Total (*n* = 103)**	**Non-ARDS cohort (*n* = 80)**	**ARDS cohort (*n* = 23)**	***P*-value**
**Demographic variables**				
Age (years)	75 (64, 87)	74 (63, 87)	84 (75, 89)	0.014
Gender, *n* (%)				0.917
Male	48 (46.6%)	38 (47.5%)	10 (43.5%)	
Female	55 (53.4%)	42 (52.5%)	13 (56.5%)	
BMI (kg/m2)	26.9 (22.7, 33.5)	26.5 (23.0, 33.9)	27.2 (24.1, 34.3)	0.346
Marital status, *n* (%)				0.269
Single	8 (7.8%)	6 (7.5%)	2 (8.7%)	
Married	93 (90.3%)	72 (90.0%)	21 (91.3%)	
Other	2 (1.9%)	2 (2.5%)	0 (0%)	
**Comorbidities**				
Congestive heart failure, *n* (%)	30 (29.1%)	23 (28.8%)	7 (30.4%)	1.000
Hypertension, *n* (%)	54 (52.4%)	41 (51.2%)	13 (56.5%)	0.834
Diabetes, *n* (%)	25 (24.3%)	18 (22.5%)	7 (30.4%)	0.613
Chronic kidney disease, *n* (%)	21 (20.4%)	15 (18.8%)	6 (26.1%)	0.634
Arrhythmia, *n* (%)	13 (12.6%)	11 (13.8%)	2 (8.7%)	0.774
Respiratory support				0.002
Spontaneous breathing, *n* (%)	38 (36.9%)	38 (47.5%)	0 (0%)	
Nasal cannula, *n* (%)	24 (23.3%)	17 (21.2%)	7 (30.4%)	
Mask ventilation, *n* (%)	7 (6.8%)	4 (5%)	3 (13%)	
High flow, *n* (%)	31 (30.1%)	19 (23.8%)	12 (52.2%)	
Non-invasive ventilator, *n* (%)	1 (0.97%)	1 (1.2%)	0 (0%)	
Intubation, *n* (%)	2 (1.94%)	1 (1.2%)	1 (4.3%)	
**Onset symptoms**				
Fever, *n* (%)	42 (40.8%)	37 (46.2%)	5 (21.7%)	0.062
Cough, *n* (%)	56 (54.4%)	43 (53.8%)	13 (56.5%)	1.000
Sore throat, *n* (%)	9 (8.7%)	9 (11.2%)	0 (0%)	0.206
Nausea, *n* (%)	2 (1.94%)	1 (1.2%)	1 (4.3%)	0.927
Headache, *n* (%)	7 (6.8%)	4 (5%)	3 (13%)	0.378
Chest distress, *n* (%)	2 (1.94%)	2 (2.5%)	0 (0%)	1.000
**Vital signs at admission**				
T (°C)	36.80 (36.50 to 37.30)	36.80 (36.50 to 37.50)	36.70 (36.55 to 37.05)	0.430
SBP (mmHg)	129.00 (111.00, 145.00)	126.09 ± 24.77	134.65 ± 26.13	0.152
DBP (mmHg)	69.00 (64.50 to 80.00)	72.11 ± 15.27	73.22 ± 13.43	0.754
HR (/min)	96.00 (81.00 to 113.50)	96.00 (80.00 to 111.00)	105.00 (85.00 to 125.00)	0.139
RR (/min)	20.00 (18.00 to 25.00)	20.00 (18.00 to 25.00)	21.00 (19.50 to 24.00)	0.293
**Aeration variables**				
PaO2 (mmHg)	89 (66, 118)	94.5 (76, 137.5)	94.5 (76, 137.5)	<0.001
PaCO2 (mmHg)	37.5 (32.5, 44.5)	37.2 (31.9, 44.5)	37.2 (31.9, 44.5)	0.358
SpO2 (%)	97 (95, 99)	98 (95, 99)	98 (95, 99)	0.022
PaO2/FiO2	192.0 (159.0, 252.0)	201.0 (171.0, 265.9)	201.0 (171.0, 265.9)	<0.001
Routine blood test			38 (34.1, 45.3)	
White blood cell (K/UL)	8.59 (6.30, 13.21)	8.16 (6.03, 13.14)	96 (91, 98)	0.152
Neutrophil (K/UL)	7.30 (4.96, 12.25)	6.36 (4.41 to 11.38)	159.5 (140.0, 171.5)	0.024
Monocyte (K/UL)	0.47 (0.32, 0.74)	0.48 (0.32 to 0.74)		0.800
Reb blood cell (K/UL)	3.46 ± 0.90	3.47 ± 0.90	10.72 (7.59, 14.26)	0.988
Platelet (K/UL)	163.00 (108.50, 256.00)	158.50 (107.50, 256.00)	9.63 (7.22 to 14.98)	0.553
Hemoglobin (g/dL)	10.41 ± 2.92	10.34 ± 2.96	10.72 (7.59, 14.26)	0.835
Glucose (mg/l)	7.10 (5.70 to 10.15)	6.65 (5.55 to 9.70)	9.63 (7.22 to 14.98)	0.023
**Inflammation**				
C-reactive protein (mg/L)	46.49 (22.49, 77.53)	38.50 (19.29 to 75.03)	0.39 (0.30 to 0.77)	0.021
Procalcitonin (ng/mL)	0.32 (0.08, 0.81)	0.26 (0.07 to 0.77)	3.46 ± 1.02	0.212
Serum Amyloid A (mg/L)	155.01 (49.84, 350.00)	100.26 (27.21 to 350.00)	186.00	0.004
**Biochemical test**				
ALT (U/L)	22.00 (11.00, 43.50)	20.00 (10.50 to 45.50)	22.00 (14.50 to 37.50)	0.590
AST (U/L)	31.00 (21.50, 48.50)	29.50 (21.50 to 49.00)	37.00 (21.00 to 47.00)	0.791
LDH (U/L)	276.00 (220.00, 88.00)	263.50 (210.00 to 388.00)	291.00 (236.50 to 388.50)	0.289
Bilirubin (mg/dl)	13.70 (9.45, 19.60)	13.75 (9.65 to 19.90)	12.20 (9.05 to 17.95)	0.571
Urea (mmol/L)	8.78 (5.37, 15.85)	7.06 (4.96 to 13.77)	15.85 (9.00 to 24.14)	<0.001
Creatine (mg/l)	79.00 (49.50, 146.50)	71.50 (48.00 to 126.00)	92.00 (60.00 to 193.00)	0.183
eGFR (ml/min)	76.00 (36.50, 95.50)	77.50 (38.00 to 102.00)	67.00 (19.50 to 86.00)	0.128
PH	7.40 (7.35 to 7.45)	7.42 (7.37 to 7.45)	7.35 (7.30 to 7.42)	0.003
Sodium (mmol/L)	139.00 (135.00, 144.00)	139.00 (134.00, 142.00)	141.00 (137.00, 151.00)	0.060
Potassium (mmol/L)	3.60 (3.10, 4.00)	3.50 (3.10, 4.00)	3.90 (3.50, 4.15)	0.112
Chlorine (mmol/L)	105.00 (99.00, 112.00)	102.50 (98.00, 110.50)	110.00 (104.00, 119.50)	0.003
Calcium (mmol/L)	1.09 (1.06, 1.14)	1.09 (1.04, 1.13)	1.12 (1.08, 1.17)	0.030
Albumin (g/dl)	2.8 (2.2, 3.6)	2.8 (2.3, 3.6)	2.7 (2.0, 3.5)	0.418
TG (mmol/L)	1.42 (0.88, 1.81)	1.48 (0.90, 1.80)	1.37 (0.68, 1.86)	0.568
TC (mmol/L)	3.36 (2.82, 4.55)	3.47 (2.82, 4.22)	3.28 (2.70, 5.35)	0.994
HDL (mmol/L)	0.86 (0.62, 1.06)	0.87 (0.59, 1.07)	0.84 (0.64, 1.01)	0.862
LDL (mmol/L)	2.30 (1.64 to 2.58)	2.30 (1.64, 2.57)	2.27 (1.90, 3.27)	0.724
Non-HDL (mmol/L)	2.47 (1.95, 3.05)	2.47 (1.95, 3.00)	2.56 (2.13, 3.73)	0.360
BNP (pg/ml)	190.00 (88.00, 492.50)	187.00 (86.00, 562.00)	195.00 (126.00, 313.50)	0.871
TNI (ng/ml)	0.04 (0.01, 0.07)	0.03 (0.01, 0.07)	0.04 (0.02, 0.07)	0.397
Mb (μg/L)	92.40 (43.50, 247.25)	81.50 (39.15, 233.25)	149.20 (87.50, 350.45)	0.021
CKMB (ng/ml)	2.50 (1.50, 5.10)	2.40 (1.40, 3.50)	4.60 (2.30, 8.10)	0.008
**Blood coagulation test**				
TT (seconds)	15.20 (14.60 to 16.40)	15.15 (14.60 to 16.45)	15.30 (14.60 to 16.10)	0.994
APTT (seconds)	31.60 (27.30 to 36.15)	31.25 (27.50 to 36.30)	32.50 (27.05 to 35.90)	0.698
PT (seconds)	12.90 (11.90 to 14.80)	12.75 (11.70 to 14.75)	13.20 (12.35 to 15.45)	0.139
INR	1.10 (1.02 to 1.27)	1.10 (1.00 to 1.27)	1.13 (1.05 to 1.33)	0.212
FG (g/L)	3.98 (3.01 to 4.61)	3.76 (2.79 to 4.58)	4.54 (3.89 to 4.72)	0.005
DD (mg/L)	1.56 (0.68 to 3.19)	1.36 (0.59 to 2.48)	2.42 (1.44 to 3.58)	0.017
FDP (mg/L)	11.90 (5.90 to 20.95)	11.15 (5.40 to 20.10)	18.10 (10.55 to 24.55)	0.040
**Lymphocyte subsets**				
Lymphocyte (10e9/L)	0.73 (0.50 to 1.01)	0.79 (0.54 to 1.26)	0.63 (0.40 to 0.76)	0.017
T lymphocyte (10e6/L)	424.30 (268.90, 679.40)	526.65 (291.05 to 858.10)	309.90 (220.00, 429.75)	<0.001
B lymphocyte (10e6/L)	82.00 (41.65, 156.60)	82.05 (38.50 to 152.35)	79.50 (44.35 to 172.65)	0.994
Th lymphocyte (10e6/L)	280.90 (154.40, 434.35)	294.20 (174.15 to 482.20)	199.40 (86.80 to 361.25)	0.025
Ts lymphocyte (10e6/L)	149.90 (88.30 to 244.50)	158.30 (97.35 to 244.50)	129.10 (48.35 to 236.15)	0.167
Natural killer cell (10e6/L)	115.60 (63.40 to 184.60)	126.00 (69.30 to 201.35)	87.60 (52.90 to 143.95)	0.033
CD4/CD8 ratio	1.60 (1.14 to 2.56)	1.58 (1.12 to 2.42)	1.60 (1.14 to 2.92)	0.669
**Cytokine profiles**				
IL1 (pg/ml)	1.22 (0.83 to 1.69)	1.22 (0.76 to 1.57)	1.37 (0.94 to 2.55)	0.224
IL2 (pg/ml)	1.03 (0.61 to 1.69)	1.03 (0.66 to 1.47)	1.06 (0.58 to 1.94)	0.571
IL4 (pg/ml)	1.45 (1.08 to 2.17)	1.35 (1.07 to 2.09)	1.67 (1.27 to 2.54)	0.132
IL5 (pg/ml)	0.79 (0.38 to 1.14)	0.76 (0.37 to 1.14)	0.97 (0.63 to 1.20)	0.226
IL6 (pg/ml)	46.91 (20.91 to 113.00)	37.58 (17.13 to 81.49)	118.00 (50.28 to 279.58)	<0.001
IL8 (pg/ml)	16.07 (6.26 to 53.18)	13.13 (5.93 to 51.21)	48.32 (14.11 to 91.98)	0.034
IL10 (pg/ml)	4.12 (2.28 to 6.26)	3.58 (2.28 to 6.14)	5.16 (2.49 to 10.01)	0.328
IL17A (pg/ml)	3.28 (1.31 to 4.58)	3.02 (1.27 to 4.42)	3.44 (1.35 to 5.58)	0.542
TNF (pg/ml)	1.98 (1.26 to 2.79)	1.90 (1.26 to 2.66)	2.48 (1.06 to 3.42)	0.169
IFN-α (pg/ml)	1.04 (0.66 to 2.06)	0.98 (0.65 to 1.69)	1.36 (0.95 to 2.60)	0.083
IFN-γ (pg/ml)	1.53 (1.11 to 1.89)	1.53 (1.11 to 1.94)	1.53 (1.14 to 1.79)	0.921

### A summary of collected CT images

Original chest CT images containing fields of the lung parenchyma were obtained from 103 patients. The total number of included CT images was 3,187, of which 690 CT slices were from COVID-19 ARDS patients and 2,497 CT slices were from non-COVID-19 ARDS patients. We manually classified 897 CT slices from 30 patients into normal CT images or abnormal CT images.

### The multivariate logistic regression analysis of clinical features

**Figure 2:**
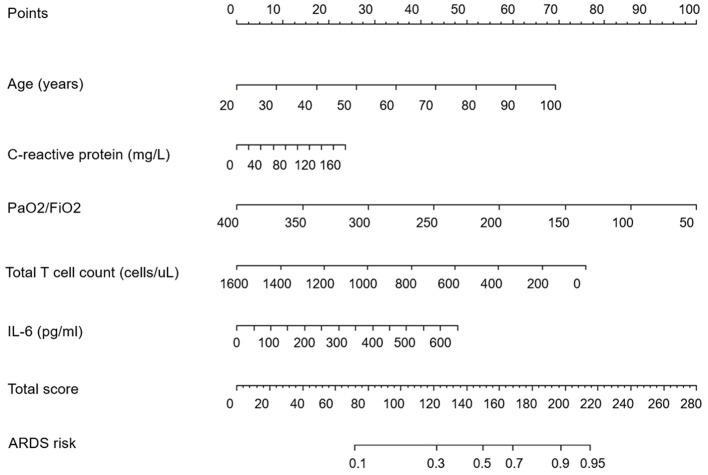
The nomogram plot for the prediction of COVID-19 ARDS.

After the multivariate logistic regression analysis, five risk factors were figured out to be independently associated with COVID-19 ARDS. We concluded that age (OR, 1.093; 95% CI, 1.015–1.177), PaO2/FiO2 ratio (OR, 0.977; 95% CI, 0.963–0.991), C-reactive protei*n* (OR, 1.017; 95% CI, 1.001–1.033), the count of total T lymphocytes (OR, 0.996; 95% CI, 0.993–0.999), and IL-6 (OR, 1.008; 95% CI, 1.002–1.017) were independent risk factors of COVID-19 ARDS. The detailed results of the multivariate logistic regression analysis are shown in Table 2. A nomogram plot was illustrated based on the result of the multivariate logistic regression model (Figure 2). We could calculate the risk score and the corresponding possibility of COVID-19 ARDS using the nomogram.

**Table 2:** Multivariate logistic regression analysis of risk factors of COVID-19 ARDS based on selected variables in the training cohort.

**Variable**	**Coefficient**	**OR (95% CI)**	***P*-value**
Age	0.089	1.093 (1.015, 1.177)	0.018
P/F ratio	−0.024	0.977 (0.963, 0.991)	0.001
CRP	0.017	1.017 (1.001, 1.033)	0.036
T lymphocyte	−0.004	0.996 (0.993, 0.999)	0.021
IL-6	0.008	1.008 (1.002, 1.017)	0.045

OR, odds ratio; CI, confidence interval.

### The predictive performance of models based on clinical features

**Figure 3:**
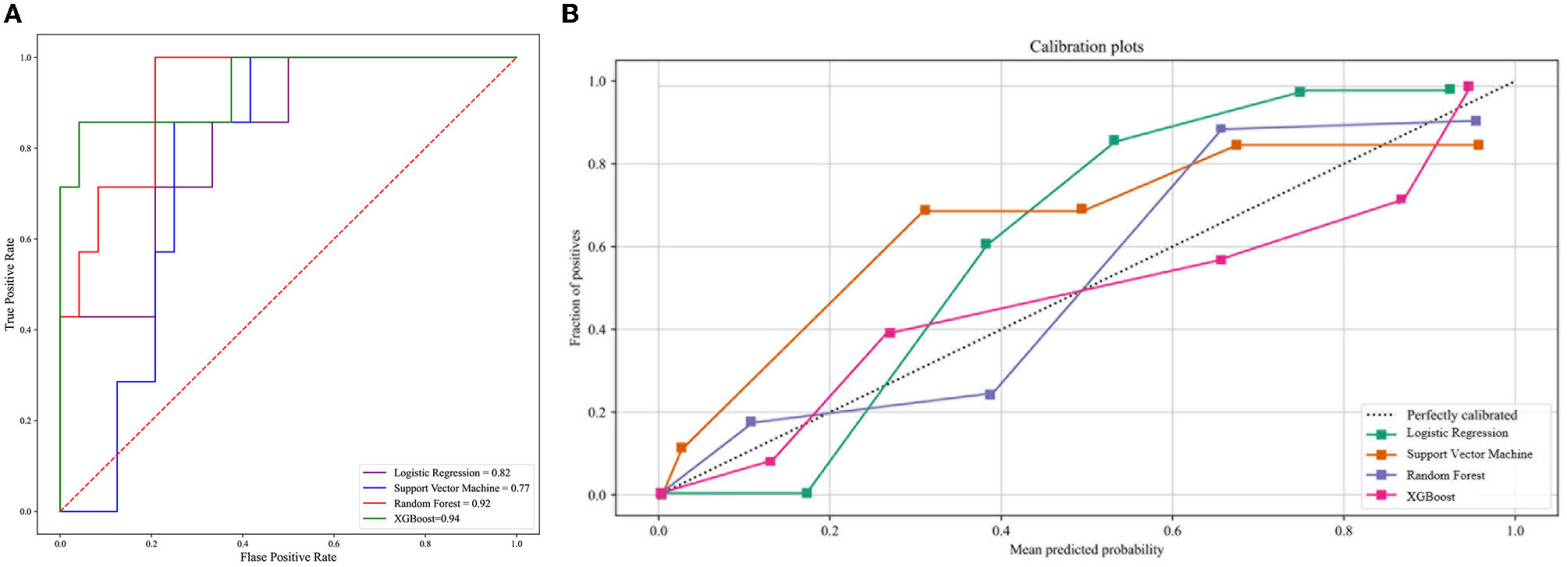
**(A)** The ROC curve of the four machine learning models. **(B)** The calibration curve of the four machine learning models.

We developed four machine learning models to predict COVID-19 ARDS, including logistic regression, support vector machine, random forest, and extreme gradient boosting. The ROC curves of all the machine learning models are shown in Figure 3A. The area under the ROC curve of the XGBoost model was 0.94, which outperformed the logistic regression model (AUC = 0.82), the support vector machine model (AUC = 0.77), and the random forest model (AUC = 0.92). We also performed the Delong test to compare the AUCs of the XGBoost model against the other three models (XGBoost model vs. logistic regression model, *P* < 0.001; XGBoost model vs. support vector machine model, *P* < 0.001; and XGBoost vs. random forest model, *P* = 0.002). The calibration curves are provided in Figure 3B. The XGBoost model was finally chosen to be the best machine learning model to predict COVID-19 ARDS in our study.

### The predictive performance of the CNN model based on CT images

**Figure 4:**
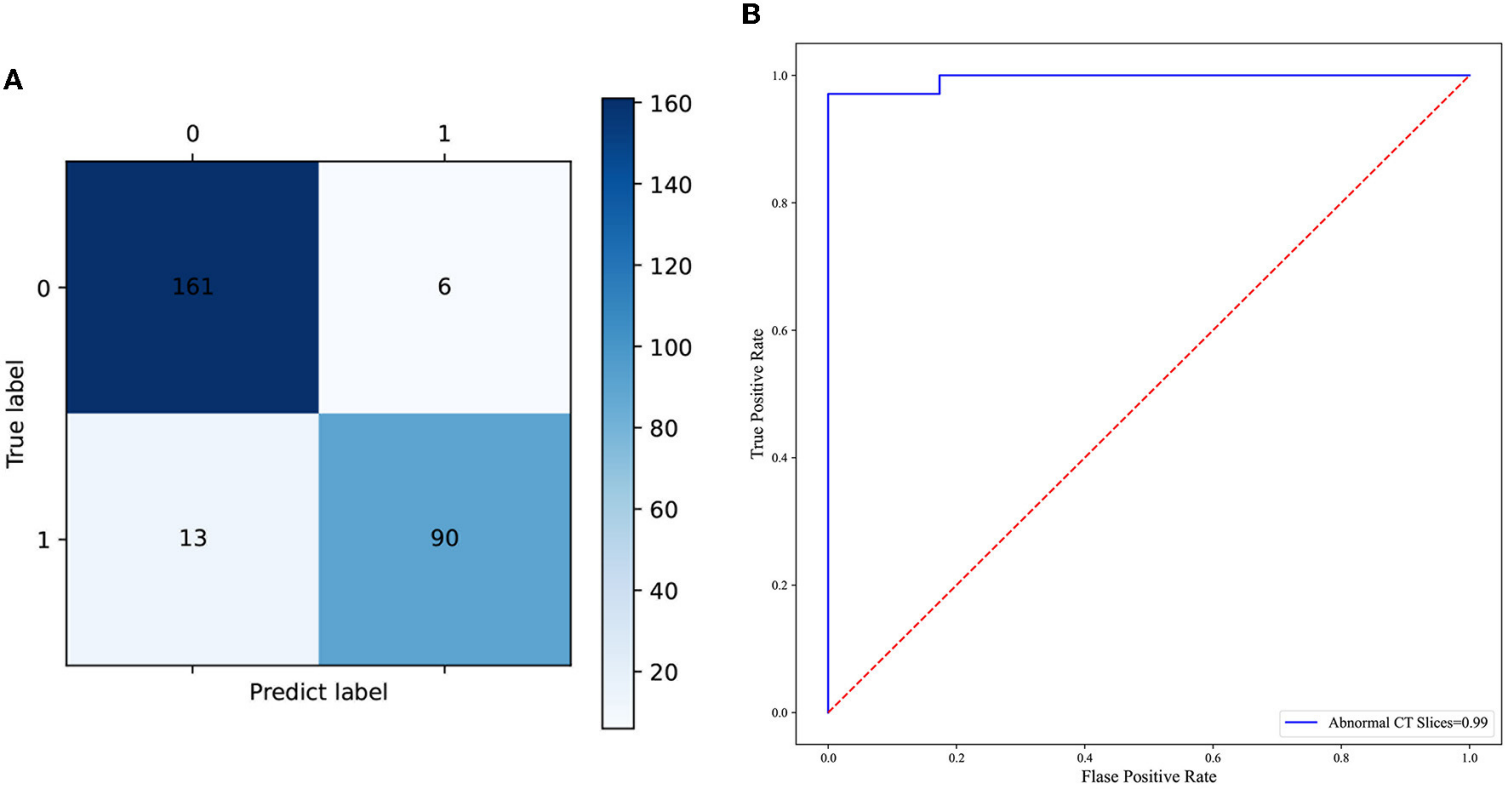
**(A)** The confusion matrix of the predictive performance of the individual CT slices classification model. **(B)** The ROC curve of the classification model of the individual CT slices.

In total, 897 manually labeled CT slices were used to train the classification CNN model based on individual CT images. Figure 4A shows the ROC curve of the classification CNN model (AUC = 0.99). The confusion matrix of the classification CNN model is shown in Figure 4B. The normal CT slices and the abnormal CT slices were correctly distinguished by the classification CNN model.

### The predictive performance of the integrated deep learning model

**Figure 5:**
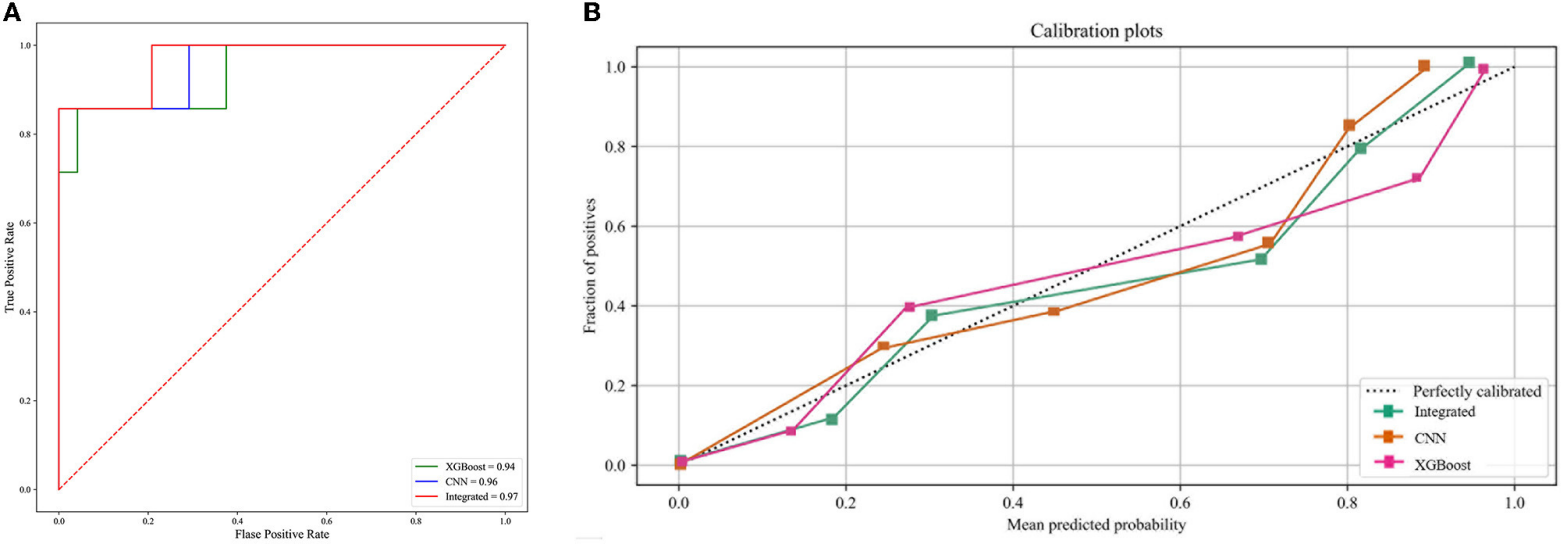
**(A)** The ROC curve of the three predictive models. **(B)** The calibration curve of the three predictive models.

The integrated deep learning model consisted of the XGBoost model based on the clinical features and the CNN model based on the selected CT slices from the individual patients. The ROC curves of the two individual models and the integrated deep learning model are shown in Figure 5A. The area under the ROC curve values of the XGBoost model, the CNN model, and the integrated model were 0.94 (95% CI: 0.91–0.96), 0.96 (95% CI: 0.93–0.98), and 0.97 (95% CI: 0.95–0.99), respectively.

**Figure 6:**
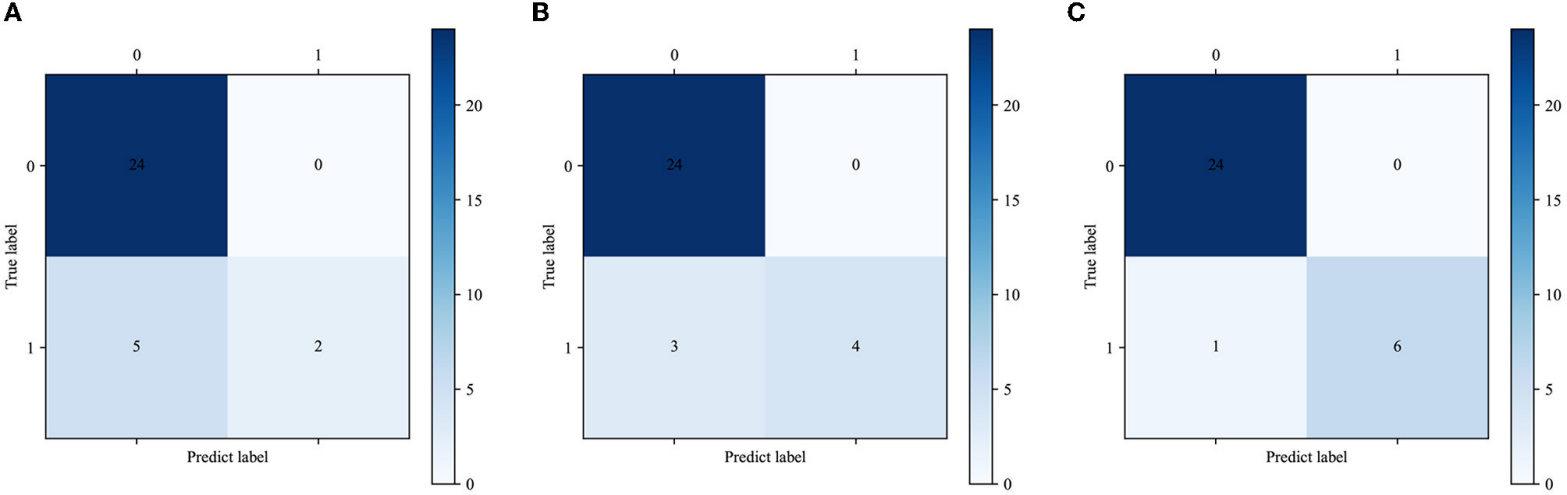
**(A)** The confusion matrix of the XGBoost model. **(B)** The confusion matrix of the CNN model. **(C)** The confusion matrix of the integrated model.

The calibration curve plot indicated a good agreement between the predicted probabilities of COVID-19 ARDS calculated by the predictive models and the actual outcome (Figure 5B). The confusion matrices were plotted using clinical features, CT images, and integrated data to predict COVID-19 ARDS (Figure 6). We found that the integrated deep learning model could yield more accurate predictions than the individual model based on clinical features or CT images. More details about the predictive performance of the models are provided in Table 3.

**Table 3:** Predictive performance of established models in validation cohort.

**Models**	**Accuracy**	**Precision**	**Sensitivity**	**Specificity**	**AUC (95% CI)**
XGB	0.84	0.29	1	0.83	0.94 (0.91, 0.96)
CNN	0.90	0.57	1	0.89	0.96 (0.93, 0.98)
XGB + CNN	0.97	0.86	1	0.96	0.97 (0.95, 0.99)

AUC, area under curve; XGB, extreme gradients boosting; CNN, convolutional neural network; CI, confidence interval.

## Discussion

The outbreak of COVID-19 led to a global pandemic, and the main causes of the deaths were pulmonary complications such as acute respiratory distress syndrome. A comprehensive analysis of the clinical symptoms, laboratory test results, and CT images is crucial to help understand the scope of COVID-19. We believe that an ensemble predictive model based on the integrated data from the patients could provide more information about the risk factors of complications such as ARDS brought on by COVID-19. Moreover, detailed and accurate risk evaluation of COVID-19 ARDS is important for clinicians to provide more personalized treatment to patients. Some published studies have applied advanced artificial intelligence methods to predict the prognosis of COVID-19 (17–20). They demonstrated the value of machine learning algorithms for predicting the outcomes of COVID-19, but no radiology information was included in the studies (21, 22). Lee et al. developed a deep learning model comprising the chest radiology score and clinical information to predict severe illness in COVID-19 patients (23). However, chest radiology is not suitable for the confirmation of diagnosis or evaluation of COVID-19 outcomes (24). Wang et al. reported an automatic quantitative model based on CT images to predict ARDS in COVID-19 patients (25). In this study, the infection fields of the lung were segmented for the quantitative analysis of the volume and density. We thought the quantitative analysis of CT images could not make the most of the CT information and thus may yield less accurate predictions.

In this retrospective study, we developed three models for the prediction of COVID-19 ARDS. Two individual models were established based on the clinical features data and the CT images, respectively; the third deep learning model was integrated by the two individual models. We found that the integrated deep learning model could offer better discriminatory performance for predicting COVID-19 ARDS than the two individual models. To strengthen the understanding of COVID-19 ARDS, we performed the multivariate logistic regression to find out the independent risk factors associated with COVID-19 ARDS and depicted the nomogram plot for it. We found that age, the concentration of c-reactive protein, PaO2/FiO2 ratio, the count of total T lymphocytes, and the level of IL-6 were related to COVID-19 ARDS. The inevitable deterioration in immunity response in senior citizens may be the reason for advanced age being a risk factor for COVID-19 ARDS (26). COVID-19 is manifested as a multisystemic disease, and the hyperinflammatory response is extremely associated with its outcome (27). COVID-19 ARDS also causes typical lung pathological changes, which are accompanied by acute and chronic inflammation (28, 29). High concentrations of CRP and IL-6 may indicate a pro-inflammatory state, which has been reported as a risk factor for a severe outcome (26, 27). It is reported that critically ill COVID-19 patients exhibited a status of immune cell hyporesponsiveness when compared to healthy people (28). Several studies have highlighted the values of T-lymphocyte subset absolute counts in predicting morbidity in COVID-19 patients (29–31). The XGBoost model was selected as the best model to handle the clinical features data because of the best predictive performance tested in the validation cohort. XGBoost stands for “Extreme Gradient Boosting” and was first proposed by Friedman (32). The XGBoost model is one of the ensembling learning algorithms, which makes precise predictions based on a series of weak classifiers, and it has been applied in many studies to deal with massive medical data.

The CT scan procedure can provide more information about the severity of lung damage and acute respiratory failure with a much faster turnaround time (2, 33). The distinctive characteristics of CT slices from COVID-19 ARDS patients could be captured by the convolutional neural network. In our study, the predictive performance of the VGG-16 model was better than that of the model based on the clinical features data. VGG architecture was first proposed by the Visual Geometry Group from Oxford and ranges from 11 to 19 layers (34). The VGG models are widely used as image classifiers or the fundamental basis of newly developed models, which also use images as input data. The VGG-16 network was first used to classify the individual CT slices into normal and abnormal images. Furthermore, the individual patient-based prediction of COVID-19 ARDS was also fulfilled by the VGG-16 network. The XGBoost model and the VGG-16 network model are complementary to each other. The predictive performance of the integrated model was superior to the individual ones. The integrated deep learning model we proposed was demonstrated to be reliable in predicting COVID-19 ARDS with high accuracy in our study. The tremendous progress made in the field of artificial intelligence facilitated the analysis of massive medical data. Our deep learning model may be one example of an automatic analysis tool that can be used for various medical data or alarming systems of adverse events in critically ill patients. Once the integrated deep learning model is fused into the information system of the hospitals, it could rapidly and correctly identify patients at high risk of COVID-19 ARDS without redundant operations.

There are some limitations in our study. First, this is a single-center retrospective study with a relatively small sample size. Second, the validation of the predictive model was only performed in the internal cohort. It is unclear whether similar predictive performance can be observed in other medical centers when our models are applied.

## Conclusion

In our study, we tried to establish different models to predict COVID-19 ARDS. We found that the models based on the clinical features or the CT images could provide accurate predictions of COVID-19 ARDS. Moreover, the integrated model combining the two individual models exhibited the best predictive performance with the highest accuracy and ROC value.

## Data availability statement

The raw data supporting the conclusions of this article will be made available by the authors, without undue reservation.

## Ethics statement

The studies involving human participants were reviewed and approved by the institutional Ethics Committees at Shanghai Renji Hospital. Written informed consent for participation was not required for this study in accordance with the national legislation and the institutional requirements. Written informed consent was not obtained from the individual(s) for the publication of any potentially identifiable images or data included in this article.

## Author contributions

YG and ZH contributed to the conception and design of the study. YZ and SQ organized the database. YZ and JF performed the statistical analysis. SM, SX, and QX wrote the first draft of the manuscript. YZ, JF, ZH, ZZ, and RT wrote sections of the manuscript. All authors contributed to the manuscript revision, read, and approved the submitted version.

## References

[1] Guan WJ Ni ZY Hu Y Liang WH Ou CQ He JX. Clinical characteristics of Coronavirus disease 2019 in China. New Eng J Med. (2020) 382:1708–20. 10.1056/NEJMoa2002032 32109013 PMC7092819

[2] Yang X Yu Y Xu J Shu H Xia J Liu H. Clinical course and outcomes of critically ill patients with SARS-CoV-2 pneumonia in Wuhan, China: a single-centered, retrospective, observational study. Lancet Respir Med. (2020) 8:475–81. 10.1016/S2213-2600(20)30079-5 32105632 PMC7102538

[3] Huang C Wang Y Li X Ren L Zhao J Hu Y. Clinical features of patients infected with 2019 novel coronavirus in Wuhan, China. Lancet. (2020) 395:497–506. 10.1016/S0140-6736(20)30183-5 31986264 PMC7159299

[4] Gibson PG Qin L Puah SH. COVID-19 acute respiratory distress syndrome (ARDS): clinical features and differences from typical pre-COVID-19 ARDS. Med J Aust. (2020) 213:54–6. 10.5694/mja2.50674 32572965 PMC7361309

[5] Williams GW Berg NK Reskallah A Yuan X Eltzschig HK. Acute respiratory distress syndrome. Anesthesiology. (2021) 134:270–82. 10.1097/ALN.0000000000003571 33016981 PMC7854846

[6] Adhikari NK Burns KE Friedrich JO Granton JT Cook DJ Meade MO. Effect of nitric oxide on oxygenation and mortality in acute lung injury: systematic review and meta-analysis. BMJ. (2007) 334:779. 10.1136/bmj.39139.716794.55 17383982 PMC1852043

[7] Fuller BM Mohr NM Skrupky L Fowler S Kollef MH Carpenter CR. The use of inhaled prostaglandins in patients with ARDS: a systematic review and meta-analysis. Chest. (2015) 147:1510–22. 10.1378/chest.14-3161 25742022 PMC4451707

[8] Adhikari NK Dellinger RP Lundin S Payen D Vallet B Gerlach H. Inhaled nitric oxide does not reduce mortality in patients with acute respiratory distress syndrome regardless of severity: systematic review and meta-analysis. Crit Care Med. (2014) 42:404–12. 10.1097/CCM.0b013e3182a27909 24132038

[9] Ioannidis J. Global perspective of COVID-19 epidemiology for a full-cycle pandemic. Eur J Clin Invest. (2020). 10.1111/eci.13423 33026101 PMC7646031

[10] Liu W Tao Z-W Wang L Yuan M-L Liu K Zhou L. Analysis of factors associated with disease outcomes in hospitalized patients with 2019 novel coronavirus disease. Chin Med J. (2020) 133:1032–8. 10.1097/CM9.0000000000000775 32118640 PMC7147279

[11] Bai Y Xia J Huang X Chen S Zhan Q. Using machine learning for the early prediction of sepsis-associated ARDS in the ICU and identification of clinical phenotypes with differential responses to treatment. Front Physiol. (2022) 13:1050849. 10.3389/fphys.2022.1050849 36579020 PMC9791185

[12] Huang L Song M Liu Y Zhang W Pei Z Liu N. Acute respiratory distress syndrome prediction score: derivation and validation. Am J Crit Care. (2021) 30:64–71. 10.4037/ajcc2021753 33385206

[13] Koski E Murphy J. AI in Healthcare. Stud Health Technol Inform. (2021) 284:295–9. 10.3233/SHTI210726 34920529

[14] Chan JF Yuan S Kok KH. A familial cluster of pneumonia associated with the 2019 novel coronavirus indicating person-to-person transmission: a study of a family cluster. Lancet. (2020) 395:514–23. 10.1016/S0140-6736(20)30154-9 31986261 PMC7159286

[15] Bai HX Hsieh B Xiong Z Halsey K Choi JW Tran TML. Performance of radiologists in differentiating COVID-19 from non-COVID-19 viral pneumonia at chest CT. Radiology. (2020) 296:E46–54. 10.1148/radiol.2020200823 32155105 PMC7233414

[16] ARDS Definition Task Force Ranieri VM Rubenfeld GD Thompson BT Ferguson ND Caldwell E. Acute respiratory distress syndrome: the Berlin definition. JAMA. (2012) 307:2526–33. 10.1001/jama.2012.5669 22797452

[17] Xu W Sun NN Gao HN Chen ZY Yang Y Ju B. Risk factors analysis of COVID-19 patients with ARDS and prediction based on machine learning. Sci Rep. (2021) 11:2933. 10.1038/s41598-021-82492-x 33536460 PMC7858607

[18] Sang S Sun R Coquet J Carmichael H Seto T Hernandez-Boussard T. Learning from past respiratory infections to predict COVID-19 outcomes: retrospective study. J Med Internet Res. (2021) 23:e23026. 10.2196/23026 33534724 PMC7901593

[19] Lee HW Yang HJ Kim H Kim U-H Kim DH Yoon SH. Deep learning with chest radiographs for making prognoses in patients with COVID-19: retrospective cohort study. J Med Internet Res. (2023) 25:e42717. 10.2196/42717 36795468 PMC9937110

[20] Wang Y Chen Y Wei Y Li M Zhang Y Zhang N. Quantitative analysis of chest CT imaging findings with the risk of ARDS in COVID-19 patients: a preliminary study. Ann Transl Med. (2020) 8:594. 10.21037/atm-20-3554 32566621 PMC7290545

[21] Chan LL Tan EK. Evidence of added value of chest CT in Coronavirus disease (COVID-19) pneumonia with initial negative RT-PCR results. Am J Roentgenol. (2020) 215:W41. 10.2214/AJR.20.23815 32559115

[22] Chen N Zhou M Dong X Qu J Gong F Han Y. Epidemiological and clinical characteristics of 99 cases of 2019 novel corona virus pneumonia Wuhan, China: a descriptive study. Lancet. (2020) 395:507–13. 10.1016/S0140-6736(20)30211-7 32007143 PMC7135076

[23] Silva MJA Ribeiro LR Gouveia MIM Marcelino BDR Santos CSD Lima KVB. Hyperinflammatory response in COVID-19: a systematic review. Viruses. (2023) 15:553. 10.3390/v15020553 36851766 PMC9962879

[24] Xu Z Shi L Wang Y Zhang J Huang L Zhang C. Pathological findings of COVID-19 associated with acute respiratory distress syndrome. Lancet Respir Med. (2020) 8:420–2. 10.1016/S2213-2600(20)30076-X 32085846 PMC7164771

[25] Tian S Xiong Y Liu H Niu L Guo J Liao M. Pathological study of the 2019 novel coronavirus disease (COVID-19) through postmortem core biopsies. Mod Pathol. (2020) 33:1007–14. 10.1038/s41379-020-0536-x 32291399 PMC7156231

[26] Li X Xu S Yu M Wang K Tao Y Zhou Y. Risk factors for severity and mortality in adult COVID-19 inpatients in Wuhan. J Allergy Clin Immunol. (2020) 146:110–8. 10.1016/j.jaci.2020.04.006 32294485 PMC7152876

[27] Forsblom E Helanne H Kortela E Silén S Meretoja A Järvinen A. Inflammation parameters predict fatal outcome in male COVID-19 patients in a low case-fatality area - a population-based registry study. Infect Dis. (2022) 54:558–71. 10.1080/23744235.2022.2055786 35353030

[28] Morrell ED Bhatraju PK Sathe NA. Chemokines, soluble PD-L1, and immune cell hyporesponsiveness are distinct features of SARS-CoV-2 critical illness. Am J Physiol Lung Cell Mol Physiol. (2022) 323:L14–26. 10.1152/ajplung.00049.2022 35608267 PMC9208434

[29] Zhang J Wang Z Wang X Hu Z Yang C Lei P. Risk factors for mortality of COVID-19 patient based on clinical course: a single center retrospective case-control study. Front Immunol. (2021) 12:581469. 10.3389/fimmu.2021.581469 33664741 PMC7920984

[30] Xiong L Zang X Feng G Zhao F. Wan, S, Zeng W, et al. Clinical characteristics and peripheral immunocyte subsets alteration of 85 COVID-19 Deaths. Aging. (2021) 13:6289–97. 10.18632/aging.202819 33711813 PMC7993687

[31] Kazancioglu S Yilmaz FM Bastug A Sakalli A Ozbay BO Buyuktarakci C. Lymphocyte subset alteration and monocyte CD4 expression reduction in patients with severe COVID-19. Viral Immunol. (2021) 34:342–51. 10.1089/vim.2020.0166 33264073

[32] Friedman JH. Greedy function approximation: a gradient boosting machine. Ann Stat. (2001) 29:1189–232. 10.1214/aos/1013203451

[33] Shi H Han X Jiang N Cao Y Alwalid O Gu J. Radiological findings from 81 patients with COVID-19 pneumonia in Wuhan, China: a descriptive study. Lancet Infect Dis. (2020) 20:425–34. 10.1016/S1473-3099(20)30086-4 32105637 PMC7159053

[34] Simonyan K Zisserman A. Very deep convolutional networks for large-scale image recognition. Computer Science. (2014). 10.48550/arXiv.1409.1556

